# Factors related to self-rated health of older adults in rural China: A study based on decision tree and logistic regression model

**DOI:** 10.3389/fpubh.2022.952714

**Published:** 2022-11-30

**Authors:** Min Zhang, Jian Rong, Song Liu, Beibei Zhang, Yaodong Zhao, Haibo Wang, Hong Ding

**Affiliations:** ^1^Department of Health Service Management, School of Health Management, Anhui Medical University, Anhui, China; ^2^Department of Scientific Research, The Second Hospital of Anhui Medical University, Anhui, China

**Keywords:** SRH, rural elderly, logistic, decision tree, influencing factors

## Abstract

**Objective:**

This study aimed to explore the related factors of self-rated health (SRH) by using decision tree and logistic regression models among older adults in rural China.

**Methods:**

Convenience sampling was employed with 1,223 enrolled respondents who met the inclusion criteria from 10 randomly selected villages in M County in China. The content of the questionnaire covered demographic characteristics, physical and mental health, sleep status, and risk of falling. The Pittsburgh Sleep Quality Index (PSQI) and the Morse Falls Risk Scale (MFS) were used to evaluate sleep status and risk of falling, respectively. The decision tree and logistic regression models were employed to analyze the related factors of SRH.

**Results:**

Notably, 817 (68.7%) subjects had good SRH. The logistic regression model showed that living standard, alcohol consumption, sleep quality, labor, hospitalization, discomfort, the number of chronic diseases, and mental health were associated with SRH (*P*-value < 0.05), while the decision tree model showed that the number of chronic diseases, sleep quality, mental health, hospitalization, gender, and drinking were associated with SRH. The sensitivity and specificity of the logistic regression model were 67.7 and 75.5%, respectively, and the area under the ROC curve was 0.789 (0.763, 0.816); the sensitivity and specificity of the decision tree model were 71.5, and 61.4% respectively, and the area under the ROC curve was 0.733 (0.703, 0.763).

**Conclusion:**

Decision tree and logistic regression models complement each other and can describe the factors related to the SRH of the elderly in rural China from different aspects. Our findings indicated that mental health, hospitalization, drinking, and sleep quality were the important associated factors.

## Introduction

China has increasingly developed into an aging society, with 264 million people aged 60 and over, accounting for 18.70% of the total population (1). At the same time, there is an inversion of population aging in urban and rural areas. The proportion of the population aged over 60 and 65 years in rural China is 23.81 and 17.72%, respectively, with 7.99 and 6.61 percentage points higher than those in urban areas (2). With the change in physiological and social roles, the prevalence of noncommunicable chronic diseases, disability, and social role function loss has increased significantly in the elderly (3). Compared with the elderly in urban locations, the elderly in rural areas have poorer medical and health conditions, material living standards, and self-care awareness (4, 5). Therefore, how to improve the health of the elderly in rural areas is one of the major challenges facing Chinese society.

Self-rated health (SRH) is an individual's subjective assessment of his or her health status and expectations (6, 7) and is also a recommended health testing indicator by the World Health Organization (8). Based on an overview of an individual's current health status and relevance to future health outcomes, SRH has been shown to predict mortality, morbidity, etc. in various countries/settings (9–11). SRH is a composite concept that reflects not only aspects of an individual's physical, psychological, and social adaptations but also health behaviors and cultural beliefs (12, 13).

Self-rated health is affected by many factors, such as socio-demographics, including gender, age, income level, and education level (14, 15). Health indicators cover factors such as disease, drug abuse, and mental health (16, 17). Studies have shown that suffering from one or more chronic diseases could be an important factor in poor SRH status (18). In addition, studies have shown that smoking, alcohol consumption, physical activity, and other daily behaviors and lifestyles were related to SRH (19). Sleep quality was significantly related to SRH, as shown by Min-Fang Hsu's study (20). Furthermore, the risk of poor SRH increases with the number of chronic diseases (NCDs), mental health symptoms, and decreased social support (18). From the perspective of etiology, the influencing factors of diseases can be divided into proximal factors that directly play a role and distal factors that indirectly play a role; thus, many of the above factors are not independent but synergistic [e.g., low physical activity + age + smoking can all lead to cardiovascular disease and therefore to low SRH (21)].

Logistic regression and decision tree models can be used to construct predictive models for influencing factors (22, 23). Although logistic regression highlights the main effects of influencing factors, it does not deal well with interactions or provide good decision recommendations. In contrast, the decision tree model breaks the traditional linear processing method by eliminating the collinearity among variables and including a series of logical decisions. Each path from the root nodes to the leaf nodes corresponds to a rule. The importance of the indicators is ranked to determine the main influencing factors. However, the classification effect varies with the number of nodes, thus it could be less stable and forbid main effect analysis. As a result, the interpretation of the results is limited. The joint use of the decision tree and logistic regression models can complement each other and improve the analysis performance.

A combination of decision tree model and logistic regression is still lacking at home and abroad to analyze the factors related to the SRH status of rural elderly. In this study, focusing on a population sample of older adults in a rural area of Anhui Province, we proposed two models to analyze the factors related to SRH in older adults to gain an in-depth understanding of the health status of older adults and provide a reference basis for improving the health and quality of life of rural older adults.

## Methods

### Study design and data collection

A cross-sectional study was carried out from July to August 2021 in Anhui, China. Anhui Province is located in the central region of China and is one of the major agricultural provinces. The population aged 60 years and above is 11.469 million, accounting for 18.79%; of which, 9.159 million are aged over 65 years, accounting for 15.01%.

Using a convenience sampling method, a total of 10 villages in two townships in M County were selected as the study sites, taking into account the different economies and populations, and the rural elderly who met the inclusion criteria were selected as the subjects. Inclusion criteria were as follows: (1) age ≥ 60 years, (2) normal hearing, clear consciousness, and basic communication skills, and (3) voluntary participation in this study. With the assistance of local village committees and village doctors, five postgraduates from Anhui Medical University who had been uniformly trained conducted a face-to-face survey with each subject. Prior to the survey, the purpose and procedures of the study were verbally presented to all respondents, and verbal consent was obtained from them. The question-and-answer technique was adopted, and the investigator filled in the questionnaire according to the answers. A total of 1,223 questionnaires were distributed in this study, and 1,189 valid questionnaires were recovered, with an effective recovery rate of 97.2% (1,189/1,223).

### Measurement of sleep quality

The Pittsburgh Sleep Quality Index (PSQI) was used to evaluate the sleep quality of the survey object in the past month. The scale contains 18 items measuring the following seven factors: sleep quality, sleep onset time, sleep duration, sleep efficiency, sleep disturbance, hypnotic medication, and daytime dysfunction. The score range for each factor is 0 to 3, and the cumulative score for each factor is the total PSQI score, with a total score range of 0 to 2l. A total score of ≤ 7 indicates good sleep quality, and >7 indicates poor sleep quality. This study verified that the Chinese version of the PSQI has good reliability and validity, with an overall reliability coefficient of 0.82 to 0.84 (24).

### Measurement of mental health

Mental health was assessed using the 12-item General Health Questionnaire (GHQ-12), which is widely used in epidemiological surveys and screening for mental disorders in community groups. The scale consists of 12 items, and each item contains the following four options: (1) not at all, (2) the same as usual, (3) more than usual, and (4) much more than usual. The GHQ-12 is scored as 0 for those who choose to answer the first two items and 1 for those who answer the last two. A total score of 3 on the GHQ-12 is used as the threshold, i.e., a score of ≥3 indicates poor mental health. The GHQ-12 of the Chinese version has good reliability and validity, with Cronbach's alpha coefficients ranging from 0.75 to 0.82, and retest correlation coefficients ranging from 0.82 to 0.85 (25).

### Measurement of risk of falling

The risk of falling was assessed using the Morse Falls Risk Scale (MFS). The scale assessment was not time-comsuming, was simple, operational, and had good reliability and validity with Cronbach's alpha coefficient of 0.891. Items and scoring criteria were as follows: history of falls (none = 0, yes = 25); 1 or more disease diagnoses (none = 0, yes = 15); use of mobility aids (none need, bed rest or nurse assistance = 0, crutches, walker, cane = 15, walking with furniture = 30); receiving medication (none = 0, yes = 20); gait (normal/immobility = 0, weakness = 10, severely frail = 20); and cognitive status (voluntary behavior = 0, no control = 15). A total score of <25 was considered low risk, 25–45 for moderate risk, and ≥45 for high risk of falling.

### Measurement of other variables

Other variables included basic demographics and health-related information. Specifically, the following were included: gender (female and male), age (60–69, 70–79, and ≥80 years), marital status (stable, unstable consisting of divorce, widowed, and unmarried), residence status (living alone living with others: living with children, relatives, caregivers, etc.), the education level (illiterate and junior high school), source of income (employment income, child support, and government subsidy), smoking (yes or no), drinking: drinking alcohol twice or more a week for 1 year (yes or no), taste for food (moderate, light, and salty), living standard (good and poor; good: self-assessed living standard is very good, good, and average; poor: self-reported poor or very poor), labor: the daily physical work of the respondents (heavy and easy), BMI (healthy, overweight, and obese; healthy: 18.5–23.9, overweight: ≥24, obese: ≥28). Information was also collected on noncommunicable diseases, physical discomfort (within 2 weeks), and hospitalization (within 1 year).

The dependent variable in this study is SRH, which was measured and analyzed in responding to the questionnaire item “How do you feel about your current health status?” The answers to this question were set to five options: “very good,” “good,” “fair,” “bad,” and “very bad.” The first three options were categorized as “good,” while the last two were categorized as “poor.”

### Statistical analysis

First, we used chi-square tests to examine differences between the different SRH groups (good and poor) and used ratios and percentages to describe the demographic characteristics of the participants.

**Figure 1:**
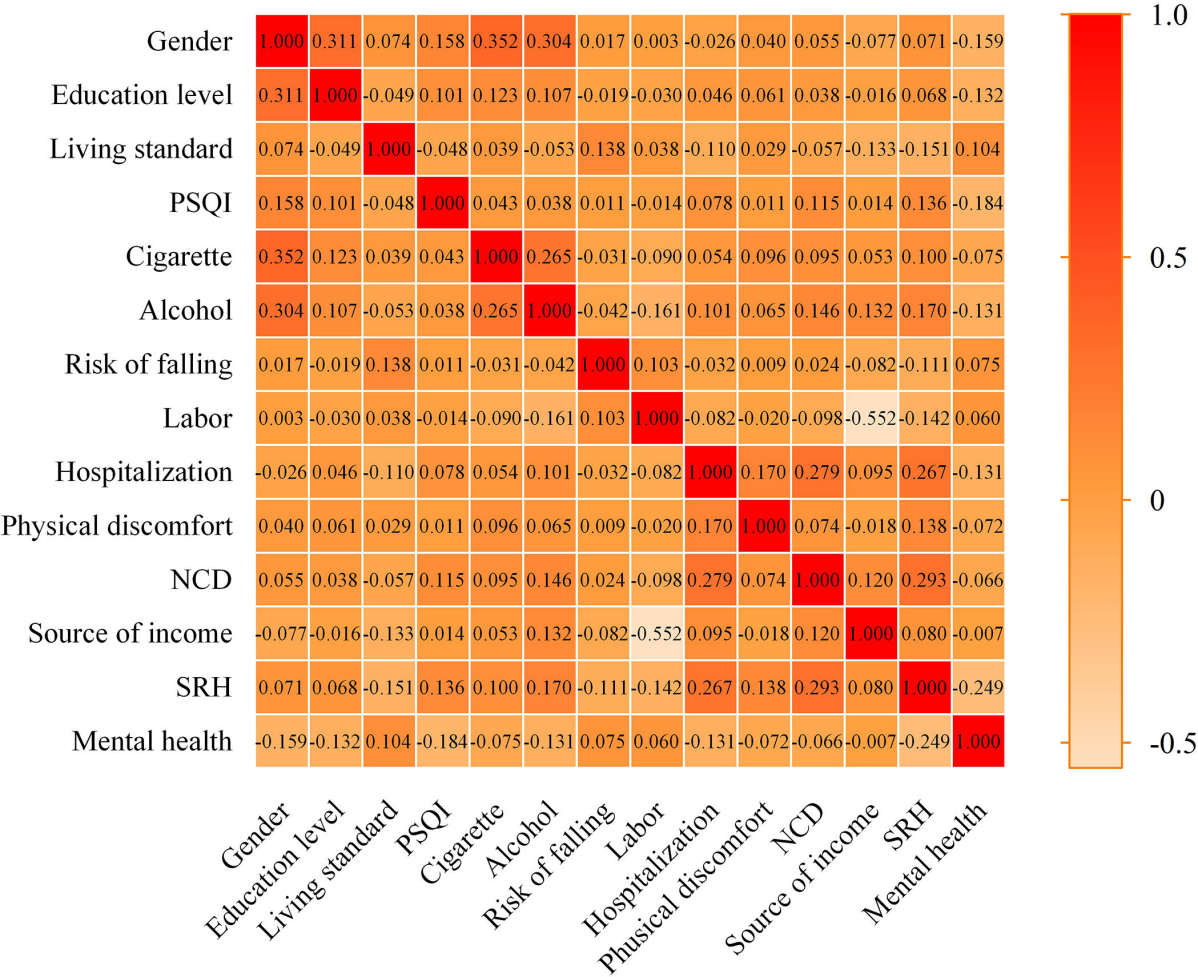
Correlation between variables.

Second, we tested correlations among the variables (Figure 1). The variables that were statistically significant in the chi-square test included logistic regression and decision tree models. To obtain the optimal model, we chose the exhaustive CHAID growth method, with a maximum growth depth of 3. The CHAID growth method uses the chi-square test or variance analysis principle to optimally segment data according to variable type and automatically judges and groups multivariate contingency tables according to *P*-value to generate a multi-tree, which can efficiently mine the main influencing factors. According to the variance expansion factor (VIF) results, there was no evidence of multicollinearity in the logistic regression model, and no factor exceeded the critical value (Supplementary Table S1).

Finally, the prediction effects of logistic regression and decision tree models were compared by constructing a subject operating characteristic curve (ROC curve), *Z*-value was calculated based on the area under the curve and standard deviation, and the corresponding *P*-value was matched to determine the difference between the two statistical models. The check level was set at α = 0.05.

All statistical analyses were performed using SPSS statistical software version 26. Two-tailed *P*-value of < 0.05 was considered statistically significant.

## Results

### Characteristics of participants

The general demographic characteristics of the respondents are shown in Table 1. A total of 1,189 subjects were included in this study, of which 817 subjects self-assessed good health. Significant statistical differences were found between the two groups on the dimensions of gender, education level, smoking, drinking, taste for food, physical labor, source of income, mental health, risk of falling, sleep quality, and NCD (*P*-value < 0.05). Among the 817 subjects, 51.8% (423/817) were female, 77.7% (635/817) did not smoke, 70.5% (576/817) did not drink, 66.6% (544/817) had not been hospitalized in the past year, and 55.3% (452/817) had economic income from their own work. At the same time, 86.3% (705/817) of the survey respondents who rated themselves as the ones having good health had a higher living standard.

**Table 1:** General characteristics of the respondents (*N* = 1,189).

	**Total** ** (*N* = 1,189)**	**SRH**		**χ^2^**	***P*-Value**
		**Good (*N* = 817)**	**Poor (*N* = 372)**		
**Gender**					
Female	602 (50.6)	394 (48.2)	208 (55.9)	6.045	0.014
**Age (years)**					
60–69	414 (34.8)	293 (35.9)	121 (32.5)	1.502	0.472
70–79	578 (48.6)	388 (47.5)	190 (51.1)		
≥80	197 (16.6)	136 (16.6)	61 (16.4)		
**Marital status**					
Stable	829 (69.7)	574 (70.3)	255 (68.5)	0.354	0.552
Unstable	360 (30.3)	243 (29.7)	117 (31.5)		
**Residence status**					
Living alone	250 (21.3)	167 (20.4)	83 (22.3)	0.539	0.463
Living with others	939 (78.7)	650 (79.6)	289 (77.7)		
**Education level**					
Illiterate	963 (81.0)	647 (79.2)	316 (84.9)	5.497	0.019
Junior high school and above	226 (19.0)	170 (20.8)	56 (15.1)		
**Living standard**					
Good	980 (82.4)	705 (86.3)	275 (73.9)	26.982	<0.001
Poor	209 (17.6)	112 (13.7)	97 (26.1)		
**Smoking**					
Yes	233 (19.6)	182 (22.3)	51 (13.7)	11.907	0.001
No	956 (80.4)	635 (77.7)	321 (86.3)		
**Alcohol**					
Yes	292 (24.6)	241 (29.5)	51 (13.7)	34.392	<0.001
No	897 (75.4)	576 (70.5)	321 (86.3)		
**Taste for food**					
Medium	523 (44.0)	370 (45.3)	153 (41.1)	4.751	0.093
Light	423 (35.6)	274 (33.5)	149 (40.1)		
Salty	243 (20.4)	173 (21.2)	70 (18.8)		
**Risk of falling**					
Low	11 (0.9)	6 (0.7)	5 (1.3)	14.986	0.001
Medium	63 (5.3)	30 (3.7)	33 (8.9)		
High	1,115 (93.8)	781 (95.6)	334 (89.8)		
**Labor**					
Heavy	652 (54.8)	487 (59.6)	165 (44.4)	24.014	<0.001
Easy	537 (45.2)	330 (40.4)	207 (55.6)		
**BMI**					
Healthy	538 (45.2)	366 (44.8)	172 (46.2)	3.122	0.210
Overweight	472 (39.7)	336 (41.1)	136 (36.6)		
Obese	179 (15.1)	115 (14.1)	64 (17.2)		
**NCD**					
0	273 (23.0)	237 (29.0)	36 (9.7)	102.672	<0.001
1	391 (32.9)	296 (36.2)	95 (25.5)		
≥2	525 (44.2)	284 (34.8)	241 (64.8)		
**Physical discomfort (within 2 weeks)**					
No	997 (83.9)	713 (87.3)	284 (76.3)	22.538	<0.001
Yes	192 (16.1)	104 (12.7)	88 (23.7)		
**Hospitalization (within a year)**					
No	686 (57.7)	544 (66.6)	142 (38.2)	84.545	<0.001
Yes	503 (42.3)	273 (33.4)	230 (61.8)		
**PSQI**					
Good	715 (60.1)	528 (64.6)	187 (50.3)	21.981	<0.001
Poor	474 (39.9)	289 (35.4)	185 (49.7)		
**Source of income**					
Labor	614 (51.6)	452 (55.3)	162 (43.5)	21.481	<0.001
Child support	360 (30.3)	214 (26.2)	146 (39.2)		
Government	215 (18.1)	151 (18.5)	64 (17.2)		
**Mental health**					
Good	859 (72.7)	652 (80.2)	207 (56.3)	73.254	<0.001
Poor	322 (27.3)	161 (19.8)	161 (43.8)		

### Results of logistic regression analysis

**Figure 2:**
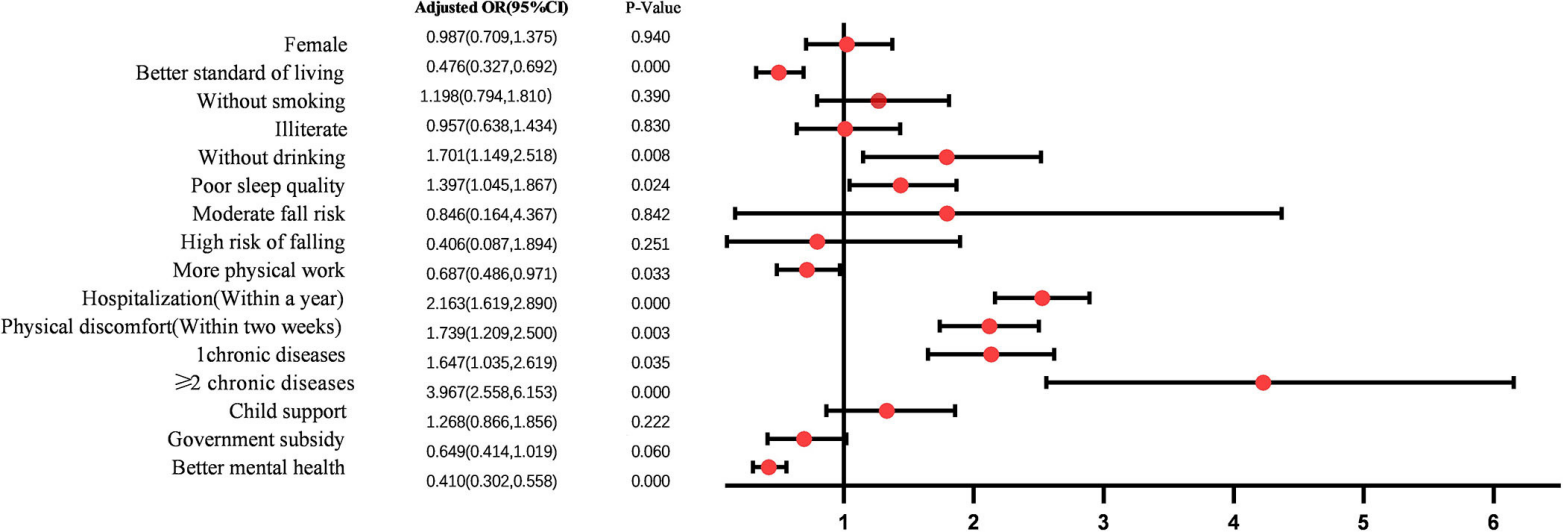
Logistic regression analysis results.

Table 2 shows the variable assignments. The results of multivariate logistic regression analysis (Figure 2) showed that living standard, drinking, PSQI, labor, hospitalization (within a year), discomfort (within 2 weeks), NCD, and mental health were influencing factors for SRH of the elderly (*P*-value < 0.05). In addition, the model did not show any statistically significant association between the risk of falling and SRH. Compared with the control group, the higher standard of living (OR = 0.476, 95% CI: 0.327–0.692), heavier physical labor (OR = 0.687, 95% CI: 0.486–0.971), and better mental health status (OR = 0.410, 95% CI: 0.302–0.558) were associated with better SRH. No drinking (OR = 1.701, 95% CI: 1.149–2.518), poor PSQI (OR = 1.397, 95% CI: 1.045–1.867), hospitalization (within a year; OR = 2.163, 95% CI: 1.619–2.890), discomfort (within 2 weeks; OR = 1.739, 95%CI: 1.209–2.500), NCD = 1 (OR = 1.647, 95%CI: 1.035–2.619), and NCD ≥ 2 (OR = 03.967, 95%CI: 2.558–6.153) were risk factors for SRH.

**Table 2:** Variable assignment table.

**Variables**	**Assignment**
Self rated health	0 = good, 1 = poor
Mental health	0 = poor, 1 = good
Gender	0 = Male, 1 = Female
Education level	0 = Junior high school and above, 1 = Illiterate
Living standard	0 = poor, 1 = good
PSQI	0 = good, 1 = poor
Smoking	0 = yes 1 = no
Alcohol	0 = yes 1 = no
Risk of falling	0 = low, 1 = medium, 2 = high
Labor	0 = easy 1 = heavy
Hospitalization (within a year)	0 = no, 1 = yes
Physical discomfort (within 2 weeks)	0 = no, 1 = yes
Chronic disease	0 = 0, 1 = 1, 2 = *d* ≥ 2
Source of income	0 = labor, 1 = child support, 2 = government subside

SRH, self-rated health; NCD, noncommunicable chronic disease; PSQI: Pittsburgh sleep quality index; GHQ-12, the 12-Item General Health Questionnaire; MFS, the Morse fall risk scale.

### Results of classification and regression tree model

**Figure 3:**
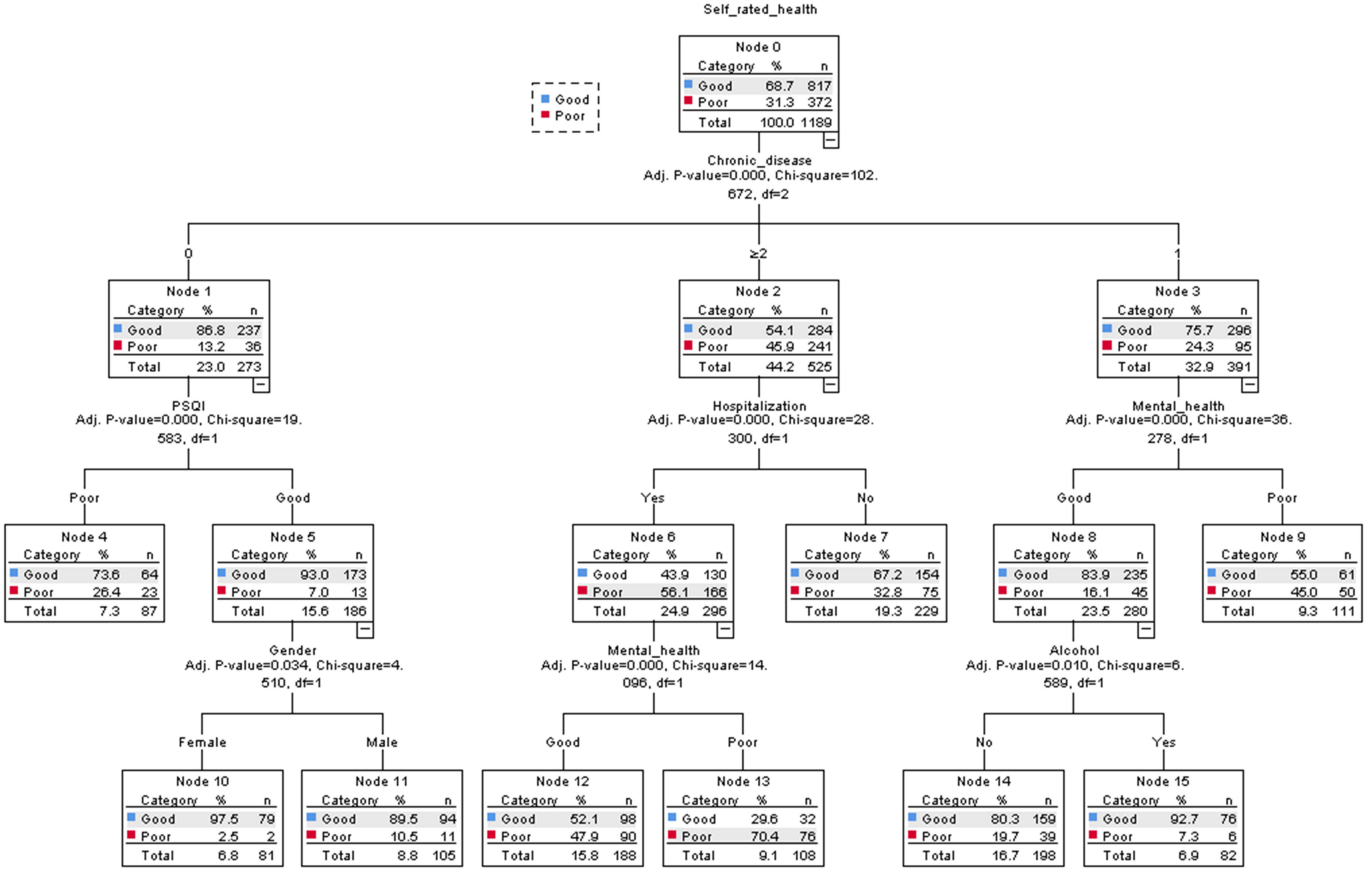
Classification and regression tree model (*N* = 1,189).

The results of the decision tree model are shown in Figure 3. SRH was mainly related to NCD, PSQI, mental health, hospitalization, gender, and drinking. NCD was the primary influencing factor for SRH. Taking NCD as the root node, the probability of having good SRH without chronic diseases in older adults is 86.85%. SRH was higher in women without chronic disease and with better sleep quality. Conversely, those with two or more chronic diseases, hospitalization within 1 year, and poor mental health have poor SRH.

### Comparison of model prediction results

**Figure 4:**
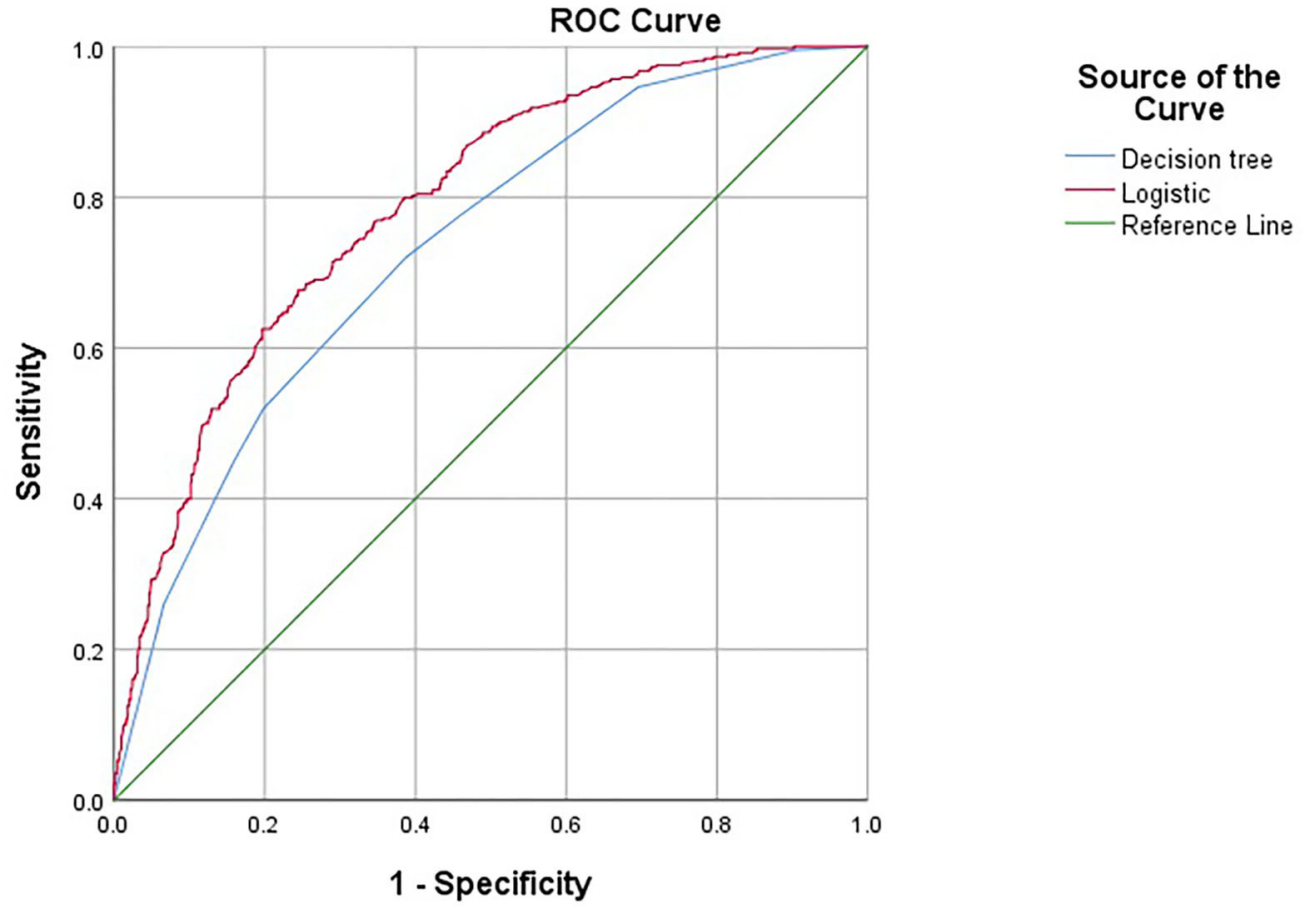
ROC curve predicted by decision tree and logistic regression model.

According to the predicted probabilities obtained by the two models as test variables, ROC curves were drawn, respectively, as shown in Figure 4. The ROC curves of both models are far away from the diagonal line, indicating that the model has a certain predictive effect. The ROC curves of both models are almost coincident, indicating that the classification effects of the two models are similar. However, it should also be noted that there are differences between the two models. The influencing factors in the logistic regression model, living standard, discomfort, and labor are eliminated in the classification decision tree model, while gender in the decision tree model is not statistically significant in the regression model.

The common influencing factors screened by the logistic regression model and decision tree model were NCD, mental health, hospitalization, drinking, and PSQI. The logistic regression model had a sensitivity of 67.7%, a specificity of 75.5%, and an area under the ROC curve of 0.789 (0.763–0.816); the decision tree model had a sensitivity of 71.5%, a specificity of 61.4%, and the area under the ROC curve was 0.733 (0.703–0.763). The difference between the two models is statistically significant (*Z* = −2.729, *P* = 0.003), and the prediction effect of the two models was moderate (0.7–0.9).

## Discussion

In this study, the prevalence of poor SRH was 31.3%. The prevalence of reported poor SRH in older adults varies in different studies. Ana M Pereira-de-Sousa found that 52.9% of Spaniards rated their health as “good” or “very good,” compared to 19% of Portuguese (*P* < 0.01) (26). A Chinese study showed that 38.33% of the study subjects reported good/excellent SRH (27). The percentage of subjects reporting good SRH in this study is somewhat higher than in the other studies discussed, possibly due to differences in national culture and historical background, as well as differences in assessment scales and samples. In general, due to age, physical function degradation, and other reasons, the elderly tend to choose a reference object in their self-assessment of health, such as relatives and friends, neighbors, and other elderly people of the same age group. Even when they are ill, they tend to adjust the reference object to maintain a more positive health self-assessment. The level of an individual's SRH depends on the selected reference group. This theory is often used to explain why people have a more positive evaluation of their own health (28).

Significant variables in the logistic regression model were different from the nodal variables that entered the decision tree model. The decision tree analysis did not reflect the effects of three factors: living standards, discomfort (within 2 weeks), and labor. The reason might be that the logistic regression model expresses the correlation of variables while the decision tree takes into account the interactions and relationships among variables and shows the function form of variables in each subcategory in detail, providing a wealth of information.

### Influence of personal characteristics on SRH

In this study, gender, age, and marital status were not significantly associated with SRH, but the proportion of women was 11.8% higher than that of men in the 372 unhealthy samples, stable marital status was higher by 37% than those with an unstable marital status, and people aged 70–79 accounted for half of the unhealthy population. Elderly people with a high standard of living, heavy physical labor, and no medical treatment within 2 weeks tend to have higher levels of SRH, which is consistent with previous studies (29, 30).

The higher SRH rates are among older adults with better living standards than those with poorer living standards, suggesting that the living standards of the elderly have a significant positive effect on their health and that improved living conditions can improve the level of SRH. An adequate source of income is an important material basis for health. On the one hand, living standards directly affect health; on the other hand, they affect health through intermediary mechanisms such as lifestyle, psychological factors, and access to health resources. As a form of social activity, physical labor can increase social opportunities for the elderly, achieve group-coordinated development, and improve SRH levels (7). Elderly people who have not seen a doctor within 2 weeks may have higher physical fitness and therefore are more likely to have higher SRH.

### NCD and SRH

Specifically, older adults who suffer from multiple chronic diseases may have lower SRH. This further confirms the consistency between the SRH and the objective state of the body. Henrike Galenkamp's study evaluated the impact of heart disease, peripheral atherosclerosis, stroke, diabetes, and other diseases on SRH and found an association between comorbidities and SRH (31). The association was nonlinear, with the effect of the individual disease being greater than the additive effect of concurrent diseases. However, starting with the second disease, the incidence of comorbid multi-morbidities was cumulatively negatively associated with SRH. At the same time, studies have shown that the elderly with multiple chronic diseases have deteriorating mental and physical health and can negatively affect SRH (32). One possible explanation for this result is that SRH in the elderly is affected by objective health, and chronic diseases bring problems such as pain, insufficient self-care ability, limitation in daily activities, and dependence on or incompatibility with drug therapy. At the same time, rural older adults with chronic diseases often require medication and hospitalization, which makes them more likely to be impoverished by their illness, thereby reducing the level of SRH. It is necessary to further improve the community supervision of chronic diseases, refine the management of chronic diseases, regularly carry out a physical examination of middle-aged and elderly people, and do a good job in community supervision of chronic diseases to further improve the health level of the population (33).

### Mental health and SRH

In short, people with goodmental health have higher SRH when compared to those with poor mental health. This is consistent with Mikyong Byun's finding that depression appeared to be the strongest predictor of negative SRH (34). Death of a spouse, chronic diseases, and reduced interpersonal communication can all lead to mental health problems. Long-term experienceof negative emotions is consistent with SRH deterioration (35). People who have been experiencing negative emotions for a long time are more likely to subjectively believe that they are in poor health due to the hypochondria effect, thus exaggerating physical health problems. People with poor mental health may suffer from dull, irritable, and even depressed moods, are tired of social communication, and lack physical activity, resulting in poorer health status.

### Hospitalization and SRH

Self-rated health tended to be more negative in older adults hospitalized within 1 year, which is consistent with the findings of Wang (36), further confirming that SRH is consistent with objective physical status (37). In this study, the elderly in rural areas were characterized by a high prevalence of chronic diseases and a low level of education. They lack knowledge about chronic diseases and their prevention and control, as well as the awareness of timely medical treatment and active use of health services (38). By the time medical care is consciously sought, the disease may have developed to a complex and severe level, therefore, SRH is lower in older adults with a history of hospitalization within 1 year. Tertiary prevention of chronic diseases to reduce their prevalence of chronic diseases and control their further development is an effective way to improve the SRH.

### Drinking and SRH

An interesting phenomenon was found in this study that drinking is a protective factor for SRH. This is consistent with the findings of Li Tongyao and Riediger (7, 39). This may be due to “Optimistic bias,” that is, drinkers believe that drinking is less harmful to them than other drinkers. In addition, it may be because drinkers are in better physical condition to tolerate a certain amount of drinking and that there are social benefits to drinking (40). At the same time, those in poor health are more inclined to avoid alcohol consumption. Moderate drinking can produce positive emotional effects and protect against cardiovascular disease, but it is not recommended to drink to improve health. In view of this, relevant departments must implement health education, gradually deepen health promotion work, effectively advocate for the elderly to develop healthy habits, quit smoking and limit alcohol, eat well, and improve levels of daily exercise.

### PSQI and SRH

Among the rural elderly population, those with better PSQI had higher SRH. This result is similar to a Japanese study of adequate sleep (COR = 5.22, *P* < 0.001), which was associated with high SRH (41). Anna Andreasson's study (42) showed that people with good sleep quality reported significantly higher SRH than those with moderate sleep quality (95% CI = 0.48, 0.89, *P* < 0.001). Insufficient sleep duration can affect circadian rhythms, impair insulin sensitivity, increase insulin resistance, decrease glucose tolerance, and lead to elevated levels of catecholamines, cortisol, and sympathetic activity, which are associated with various diseases (43). Also, poor sleep quality may increase daytime fatigue, leading to negative events and negative emotions, which can affect SRH levels. Attention should be paid to the role of sleep quality in promoting the health of the elderly. Through psychological counseling, knowledge explanation, and other means, the elderly can be helped to develop good sleep habits. Attention should always be paid to helping a pleasant mental state be maintained in order to improve physical and mental health.

### Strengths and limitations

Our study has the following advantages. First, the effective response rate in this study was 97.2% (1,189/1,223), and it is known that the results of studies with a highly effective response rates are more reliable. Second, the combined use of decision tree and logistic regression models is beneficial, where each model can be learned from and where the strengths of each one can complement the other. The combined use of both can allow analysis of the factors affecting the SRH of the elderly at different levels and help to quickly find the most influential combination of factors. Taking advantage of the intuitive effect of the decision tree, ease of interpretation, and generation of partial classification rules, combined with the logistic regression model giving parameter estimates and hypothesis testing results for each variable, the main effect variables were filtered out through the logistic regression model, and then using the decision tree, the model further investigated the interactions between the variables.

However, the limitation of this study is that the selected samples were only from the rural elderly population in 10 villages of M county. In addition, this is a cross-sectional study with a limited inference of causality, which needs to be verified by a high-quality cohort study. At the same time, our study did not include all the potential factors of SRH, which will be considered in future studies.

## Conclusion

The SRH of the rural elderly is affected by many factors such as personal characteristics, health level, behavior, and lifestyle. This study combined logistic regression and decision tree models to screen key influencing factors affecting SRH in older adults, including NCD, mental health, hospitalization, alcohol consumption, and PSQI. This study may be helpful to plan and take measures to improve SRH in older adults and promote active aging. Grass-roots medical and health institutions in rural areas should make full use of existing health resources to improve the community health care network and strengthen the rehabilitation and treatment of patients with chronic diseases, which is conducive to the improvement of the health status of the elderly in rural areas. With an increase in age, the elderly pay more attention to social environment and the degree of psychological and emotional pleasure; thus society and families should provide more care to the elderly to help them achieve healthy aging.

## Data availability statement

The original contributions presented in the study are included in the article/Supplementary material, further inquiries can be directed to the corresponding author.

## Ethics statement

All participants in this study were fully informed about the purpose and methods of this study. The purpose and procedures of the survey were explained to all respondents prior to conducting the survey, and informed consent for the survey was secured from all respondents. For illiterate respondents, informed consent was also obtained from the guardians.

## Author contributions

MZ conceptualized the study and wrote the manuscript. JR, SL, and BZ contributed to the study design, data collection, data processing, and statistical analysis. JR contributed to the literature review. MZ, JR, YZ, and HW revised the manuscript. All authors reviewed the manuscript and approved the final manuscript.

## Funding

This research was funded by the Research Projects of Humanities and Social Sciences in Colleges and Universities of Anhui Province (No. SK2018A0165) and the Doctoral Fund Project of Anhui Medical University (No. XJ201545).

## Conflict of interest

The authors declare that the research was conducted in the absence of any commercial or financial relationships that could be construed as a potential conflict of interest.

## Publisher's note

All claims expressed in this article are solely those of the authors and do not necessarily represent those of their affiliated organizations, or those of the publisher, the editors and the reviewers. Any product that may be evaluated in this article, or claim that may be made by its manufacturer, is not guaranteed or endorsed by the publisher.
