# Supplementary material for: Factors related to self-rated health of older adults in rural China: A study based on decision tree and logistic regression model
Source: Front Public Health. 2022 Nov 30;10:952714. doi: 10.3389/fpubh.2022.952714 (PMC9748102; doi:10.3389/fpubh.2022.952714)
Supplement: Supplementary file 1 [file Data_Sheet_1.docx]

| TableS1 Results of multicollinearity examination of variables | | | | | | | | |
| --- | --- | --- | --- | --- | --- | --- | --- | --- |
| **variables** | | Non-standardized coefficient | | Standard coefficient | t-Value | Sig. | Collinearity statistics | |
|  |  | B | Standard error |  |  |  | Tolerance | VIF |
|  | (constant) | .397 | .111 |  | 3.578 | .000 |  |  |
|  | Gender | .038 | .030 | .039 | 1.263 | .207 | .713 | 1.402 |
|  | Education level | -.051 | .035 | -.041 | -1.456 | .146 | .876 | 1.142 |
|  | Living standard | .078 | .036 | .060 | 2.181 | .029 | .919 | 1.088 |
|  | PSQI | -.041 | .027 | -.041 | -1.506 | .132 | .930 | 1.076 |
|  | Cigarette | -.039 | .036 | -.031 | -1.091 | .276 | .833 | 1.200 |
|  | Alcohol | -.011 | .033 | -.010 | -.336 | .737 | .830 | 1.205 |
|  | Risk of falling | .080 | .047 | .045 | 1.700 | 3089 | .963 | 1.038 |
|  | Labor | .001 | .031 | .001 | .047 | .963 | .699 | 1.431 |
|  | Hospitalization | -.136 | .028 | -.137 | -4.879 | .000 | .867 | 1.153 |
|  | Physical discomfort | -.042 | .036 | -.031 | -1.173 | .241 | .955 | 1.047 |
|  | Chronic disease | -.184 | .017 | -.296 | -10.588 | .000 | .884 | 1.132 |
|  | Source of income | .037 | .020 | .058 | 1.831 | .067 | .685 | 1.460 |
|  | Mental health | .172 | .030 | .157 | 5.684 | .000 | .908 | 1.101 |
| a. Dependent variable\:SRH | | | | | | | | |
